# Effect of the Operational Conditions in the Characteristics of Ceramic Foams Obtained from Quartz and Sodium Silicate

**DOI:** 10.3390/ma13081806

**Published:** 2020-04-11

**Authors:** Lina Uribe, Juan D. Giraldo, Alejandro Vargas

**Affiliations:** Escuela de Ingeniería Civil de Minas, Universidad de Talca, Talca 3460000, Chile; juan.giraldo@utalca.cl (J.D.G.); avargas13@alumnos.utalca.cl (A.V.)

**Keywords:** ceramic foam, quartz, sodium silicate, lime, sodium carbonate

## Abstract

Ceramic foams were fabricated without using melting pots through the direct foaming of compacted powder mixtures of commercial quartz (SiO_2_) with fluxing agents (Na_2_CO_3_ and CaO) and a foaming agent (Na_2_SiO_3_·5H_2_O) at a relatively low temperature range (850−870 °C). The effects of the pressing pressure of the powders, the foaming time, foaming temperature, and mixture content were evaluated. The obtained cellular solid materials presented an acceptable volumetric expansion at a pressing pressure of 4 t. The materials only presented porosity at a minimum temperature of 850 °C and at a minimum time of 30 min. All the foamed samples showed an acceptable symmetric expansion and non-appreciable fissures. The study of the mixture content through the statistical software MODDE® shows that the porosity of the samples was principally affected by the Na_2_SiO_3_ content and the foaming temperature. The samples obtained at the optimum controlling factors proposed by this statistical software presented an apparent density, porosity, and mechanical strength of 1.09 ± 0.03 g/cm^3^, 56.01% ± 1.12%, and 3.90 ± 0.16 MPa, respectively. Glass and ceramics foams such as those obtained in this work become attractive as insulation materials in applications where high temperatures occur due to their higher melting points.

## 1. Introduction

Ceramic materials with the presence of porosity with any fraction, shape and size can be considered as cellular ceramics [1]. Different methodologies to obtain cellular ceramics have been reviewed [1,2]. Studart et al. have classified the most common processing routes to elaborate macroporous ceramics into three categories: direct foaming, replica, and sacrificial template methods [2]. The former is the most straightforward category for the preparation of cellular ceramics. Within this category, the sintering of powder mixtures that incorporate a foaming agent has been the most common processing route to make these porous ceramic materials, because the porosity and the pore structure can be easily controlled by adjusting the initial composition of the powder mixture [3,4]. The powder mixture is heated above its softening temperature, resulting in the sintering of a solid cellular ceramic because of the release of gas from the foaming agent.

These ceramic foams have a wide range of applications, especially for high temperatures and corrosive environments, such as separation process (filtration), thermal insulation, and support for catalysis reactions, due to their unique properties such as low densities, low thermal conductivity, thermal-shock resistance, and excellent dielectric properties [5]. However, their use has been limited to industrial applications because of the high cost of production associated with the high energy consumption and the elevated price of raw materials [6]. In recent years, another disadvantage to the manufacture of cellular ceramics is the use of foaming agents that generate environmental critical gases (e.g., CO, CO_2_, SO_2_), such as carbon and sulfated based agents [7].

In order to overcome these drawbacks to the production of glass-ceramic foams, it is necessary to use inexpensive raw materials, diminish the energy consumption, and use alternative foaming agents that do not generate environmental critical gases through oxidation reactions or thermal decomposition [2,7,8,9,10,11]. One inexpensive raw material used to produce cellular ceramics is the conventional quartz [12]. The mixture of quartz with Na_2_CO_3_ and CaO as fluxing agents [1,3,4] and Na_2_SiO_3_·5H_2_O as a foaming agent [13] could be directly foamed using a powder foaming method at low temperatures (800–900 °C) reducing the production of environmental critical gases and the cost of the production process [14]. Hesky et al. [7] classified hydrated sodium silicate as an environmentally friendly foaming agent, since during the foaming process, it releases water vapor instead of carbon dioxide. This study aims to research the feasibility of obtaining symmetric silica foams using compacted mixtures of these raw materials at low temperatures without using melting pots and evaluate the effect of the foaming temperature, pressing pressure, foaming time, and powder mix composition on the characteristics of quartz foams obtained in the presence of sodium silicate as an environmentally friendly foaming agent.

## 2. Materials and Methods

### 2.1. Equipment

A disk pulverizer, Bico (Burbarnk, CA, USA) and sieve shaker RX-29-10, Ro-Tap (W.S.Tyler, Mentor OH, USA) were used to prepare a sample of quartz with a size below mesh #200 (74 µm) in order to make a granulometric analysis of this sample. A semi-analytical balance BXX22, Boeco, and overhead stirrer OSD-20, Boeco (Hamburg, Germany) coupled with a Teflon impeller (4-flat-blade), hydraulic press TY12002, Torin (Ontario, CA, USA) and muffle MF10-12G, Biobase (Shandong, China) were employed to elaborate the ceramic foams. Finally, a 50 mL Pycnometer, XRF analyzer Niton XL3t, Thermo Fisher Scientific (Waltham, MA, USA), and CBR loading press 34-T0102/A (Milan, Italy) were used for the quartz and foam characterization.

### 2.2. Materials

**Figure 1:**
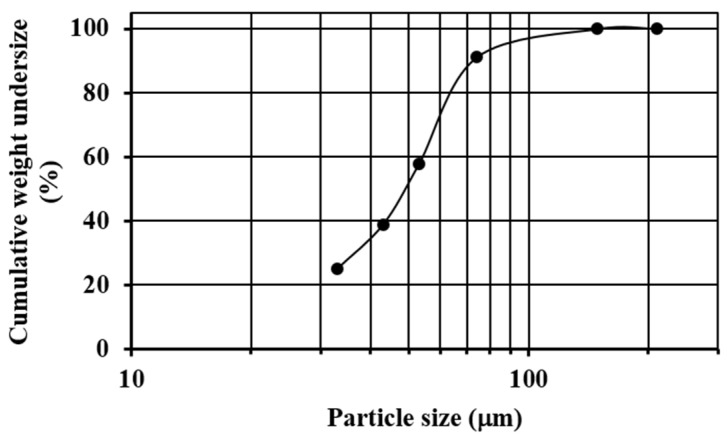
Sieve analysis of the pulverized quartz.

Commercial quartz (SiO_2_) with a particle size in the range of 3–5 mm was pulverized and sieved until almost all of the sample was below mesh #200 (74 µm). The sieving process was carried out using ASTM sieves (#70, #100, #200, #270, #325 and #400) and a sieve shaker (Ro-Tap). Figure 1 shows the granulometric analysis of the pulverized quartz, where it is observed that the median size (X50) of the sample was about 50 µm, the 80% passing size (X80) was about 70 µm, and the 33.2%w of the material was retained on mesh #270 (53 µm). 

Table 1 presents the X-ray fluorescence analysis (FRX) realized for the obtained pulverized quartz. Data shows that the sample is mainly composed of silicon. A quartz sample purely composed of SiO_2_ has an elemental composition of 46.74% of Si and 53.26% of O_2_. The presence of the other elements and the over-content of silicon indicate the existence of other mineral phases as complex silicates. 

Sodium silicate pentahydrate (Na_2_SiO_3_·5H_2_O) was used as a foaming agent, and sodium carbonate (Na_2_CO_3_) and calcium oxide (CaO) were used as fluxing agents. All materials were purchased from Winkler (Santiago, Chile) with exception of the calcium oxide that was purchased from Dideval (Santiago, Chile). Distilled water was used for all of the experiments in this work.

### 2.3. Process of Elaboration of Ceramic Foams

Powder quartz was first mechanically blended with solid fluxing agents (Na_2_CO_3_ and CaO) for 5 min. Then an aqueous solution of the foaming agent Na_2_SiO_3_·5H_2_O (200 g/L) was added slowly to the solid blend under continuous stirring for 15 min. The wet mixture was poured into a steel mold (4 cm × 4 cm × 7 cm) and pressed for 3 min. This compacted mixture (no melting pot) was sintered and foamed at a determined temperature with a sintering rate of 8.33 °C/min, and then cooled. The cooling rate from the foaming temperature to 600 °C was 5.55 °C/min. From 600 °C, the foams were cooled at room temperature. To determine the minimum time, temperature, and pressing pressure, at which porosity and a symmetric expansion of the foamed materials are produced, these factors were varied according to Table 2.

With these minimum values established, the effect of the powder mix composition and temperature on the porosity formation were evaluated according to an experimental design carried out with MODDE® (Umetrics, Sweden). The ranges and fixed values of this experimental design are shown in Table 3.

### 2.4. Ceramic Foams Characterization

The obtained cellular solids were characterized determining their apparent density $\left(\rho_{app}\right)$, real density $\left(\rho_{r}\right)$, porosity percentage (ϕ), volumetric expansion $\left(V_{e}\right)$, loss of weight (∆W), mean pore size ($A_{p}$), and estimated pore diameter ($D_{p}$). 

The apparent density was calculated with the weight and volume of a cubic piece of each cellular solid according to Equation (1).

(1) $$\rho_{app}=\frac{W_{c}}{V_{c}}$$

where $W_{c}$ and $V_{c}$ are the weight and volume of a cubic piece. To calculate the real density, this cubic piece was pulverized, and the powder density was measured using a pycnometer according to Equation (2).

(2) $$\rho_{r}=\frac{W_{pp}-W_{ep}}{\left(W_{pw}-W_{ep}\right)-\left(W_{ppw}-W_{pp}\right)}$$

where $W_{ep}$ is the weight of the empty pycnometer, $W_{pp}$ the weight of the empty pycnometer plus powder, $W_{pw}$ weight of the empty pycnometer plus water, and $W_{ppw}$ the weight of the empty pycnometer plus powder and water. Porosity was calculated according to Equation (3).

(3) $$\phi =\left(1-\frac{\rho_{app}}{\rho_{r}}\right)\times 100$$

The volumetric expansion and loss of weight of the cellular solids were calculated according to Equations (4) and (5).

(4) $$V_{e}=\frac{V_{f}-V_{i}}{V_{i}}\times 100$$

(5) $$\Delta W=W_{i}-W_{f}$$

where $V_{i}$ and $W_{i}$ are the volume and weight of the compacted mixture, and $V_{f}$ and $W_{f}$ are the volume and weight of the sintered and foamed materials. The measurements of the characteristic parameters of the samples obtained according to Table 2 were done in duplicate.

### 2.5. Computational Tools

MODDE® was used to make a multilevel quadratic design in order to evaluate the effect of the mixture composition and the temperature on the structure of the foamed materials. Considering the ranges and fixed values from Table 3, a total of twenty-six experiments were proposed, whose values are shown in detail in Table 4. As can be seen in the table, four experiments are identical (temperature of 860 °C) in order to check if the experimental design was proposed correctly and to calculate the standard deviation (SD). All the controlled factors and responses were simultaneously calibrated using partial least squares regression (PLSC). 

**Figure 2:**
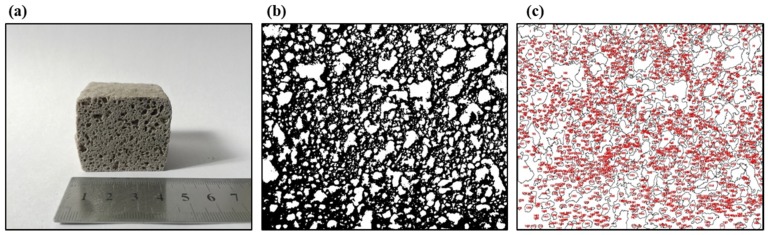
The internal structure of the foamed material at 850 °C for 45 min using sodium silicate (Na_2_SiO_3_) as a foaming agent with a mix composition of 70.3%w SiO_2_(s), 16%w Na_2_CO_3_(s), 5.4%w CaO(s), and 8.3%w Na_2_SiO_3_(l): (**a**) material scale, (**b**) image threshold, and (**c**) pore analysis using ImageJ®.

In addition, ImageJ® (National Institutes of Health, Bethesda, MD, USA) was used to estimate the mean pore size of the foamed materials. First, images of the cross-sections of the foams with a calibration scale (a rule) were opened. A straight line onto the rule with a defined distance was drawn and was selected to set the scale (see Figure 2a). Then, the images were transformed to 8-bit grayscale images, and the thresholding adjustment was applied to them. This adjustment allows the images to be divided into two classes of pixels, foreground and background (structure (black) and pores (white)) (see Figure 2b,c). These thresholded images were analyzed using the “Analyze Particles” command, giving out the mean pore size of the foamed materials ($A_{p}$, mm^2^). This mean pore size was transformed according to Equation (6) in the estimated pore diameter of the foams ($D_{p}$), assuming pores with circular cross-sections.

(6) $$D_{p}=2\sqrt{\frac{A_{p}}{\pi }}$$

## 3. Results and Discussion

### 3.1. Minimum Temperature and Time 

**Figure 3:**
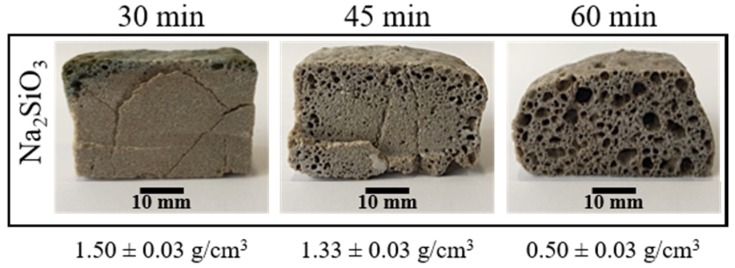
Internal structure and apparent density of the foamed and sintered materials using sodium silicate (Na_2_SiO_3_) as foaming agent (4 t by 3 min). Materials have a composition of 59.2%w SiO_2_(s), 19%w Na_2_CO_3_(s), 5.4%w CaO(s), and 16.4%w Na_2_SiO_3_(l).

To determine the lowest temperature at which porosity appears, compacted mixtures were sintered and foamed at 750, 800 and 850 °C for 30 min. The internal structure of the obtained materials only presented porosity at 850 °C. However, these porosities do not appear on all the cross-sections, and they were located principally near to the borders of the samples (see Figure 3). To achieve a porosity in all the internal structure, the time of the direct foaming process was varied from 30 to 60 min. Figure 3 shows that an increase in the time allows a porosity on all the cross-sections of the materials obtained. Figure 3 also shows how the apparent density of the materials obtained decreases with an increase in the foaming time. Although an increase in foaming time favors the appearance of porosity, samples with a foaming time of 60 min presented a less symmetric expansion and less homogeneous porosity.

### 3.2. Effect of the Pressing Pressure

**Figure 4:**
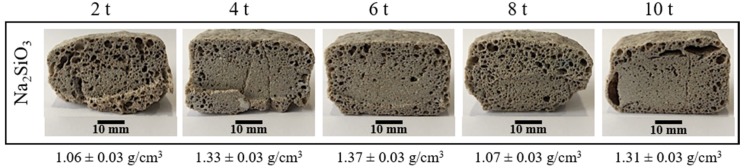
Internal structure and apparent density of the foamed and sintered materials for 45 min at 850 °C using sodium silicate (Na_2_SiO_3_) as foaming agent and varied pressing pressure. Materials have a composition of 59.2%w SiO_2_(s), 19%w Na_2_CO_3_(s), 5.4%w CaO(s), and 16.4%w Na_2_SiO_3_(l).

To see the effect of the pressing pressure on the porosity, the foaming temperature and time were fixed at 850 °C and 45 min, respectively. Figure 4 shows the effect of the pressing pressure on the porosity of the materials obtained. Visually, there is not a clear tendency on the pore formation on the entire internal structure, but it is possible to see that at the lowest pressing pressure (2 t), the sample results are more porous. However, the porosity is non homogenous with bigger pores on the edges. In addition, in the extreme values of the pressing pressure (2, 8 and 10 t), all the samples have pronounced fissures and a non-symmetric expansion. Finally, in the range of 4–6 t, although the samples presented fewer fissures with a better symmetric expansion, pores are only well formed on the edges.

**Figure 5:**
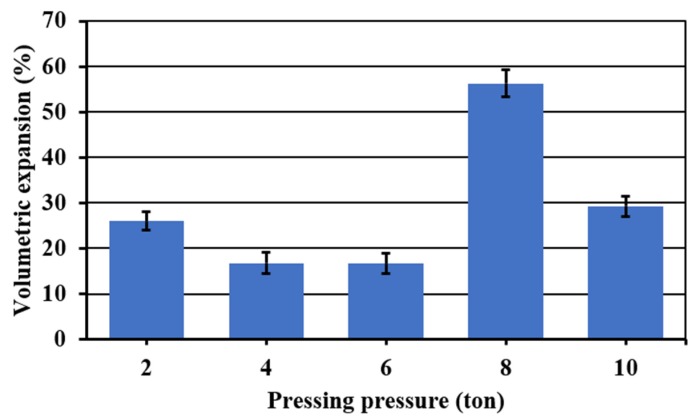
Volumetric expansion of the foamed and sintered materials for 45 min at 850 °C using sodium silicate (Na_2_SiO_3_) as foaming agent and varied pressing pressure. Materials have a composition of 59.2%w SiO_2_(s), 19%w Na_2_CO_3_(s), 5.4%w CaO(s), and 16.4%w Na_2_SiO_3_(l).

Figure 5 shows the volumetric expansion of the samples as a function of the pressing pressure. Samples in extreme values of the pressing pressure (2, 8 and 10 t) showed the higher volumetric expansions. However, at these pressing pressures, all the samples were irregularly expanded and presented fissures. Acceptable symmetric volumetric expansions without considerable fissures were obtained in a range of pressing pressure of 4–6 t. In this range, a lower apparent density was achieved at 4 t, and given that at this pressure, less wear of the press and a shorter operating time are obtained, for that reason, this one was fixed as the optimum pressing pressure.

### 3.3. Effect of the Powder Mix Composition and Temperature

Table A1 shows the results obtained in the experiments proposed using MODDE®. In total, 26 samples were prepared with varying powder mix composition and foaming temperature. Variables such as apparent density ($\rho_{app}$), porosity (ϕ), volumetric expansion ($V_{e}$), and pore diameter ($D_{p}$) were calculated and are shown in the table. In addition, the pore structure of each sample is presented. The ranges obtained for each of the response variables are shown in Table 5.

From Table A1, by comparing the essays made at 850 °C, it can be observed that samples SS-02 and SS-05, both samples with medium content of sodium silicate (16.4 and 17.0%w, respectively) and higher concentrations of sodium carbonate (19.0%w), presented the lowest apparent density (0.85 ± 0.03 and 0.83 ± 0.03 g/cm^3^, respectively) and higher volumetric expansion (137.66% ± 3.08% and 124.45% ± 3.08%, respectively). The cell structure of both samples was open, and the pore size was bigger (see Table A1). It has been reported in previous works that Na_2_CO_3_ can be used as a foaming agent as well. A big expansion with an asymmetric cell structure can be observed in the obtained results which may be related to the use of Na_2_CO_3_ and Na_2_SiO_3_. At elevated temperatures, the foaming agent releases gas when it reacts with the glass melt or decomposes due to thermal instability. If the foaming agent is homogenously distributed in the glass powder mixture, the gas formation leads to the expansion and coalescence of many small bubbles with increasing temperature and time [3]. On the other hand, at 870 °C, the samples presented a lower density (0.66–1.19 ± 0.03 g/cm^3^), higher porosity (56.98%–73.41% ± 2.13%) and high volumetric expansion (22.77%–160.42% ± 3.08%), the SS-19 assay being the sample with the lowest apparent density (0.66 ± 0.03 g/cm^3^). In addition, at 860 °C, by using medium quantities of quartz (64.75%w), sodium silicate (12.50%w), and sodium carbonate (17.5%w) with high quantities of calcium oxide (5.25%w), a medium pore diameter (0.34–0.53 ± 0.08 mm) and porosity is generated.

These response variables (apparent density ($\rho_{app}$), porosity (ϕ), volumetric expansion ($V_{e}$) and pore diameter ($D_{p}$) ) and controlled factors (powder mix composition and foaming temperature) were simultaneously calibrated using partial least squares regression through the software MODDE® to observe how the controlling factors affect these response variables directly joined to the porosity formation on the materials. According the results obtained, the variation of the mass fraction of the Na_2_CO_3_(s) and CaO(s) in the ranges evaluated did not have a representative impact on the response variables when lower contents of sodium silicate were used. The main factors that modified the porosity were the Na_2_SiO_3_(l) mass fraction and the foaming temperature. 

**Figure 6:**
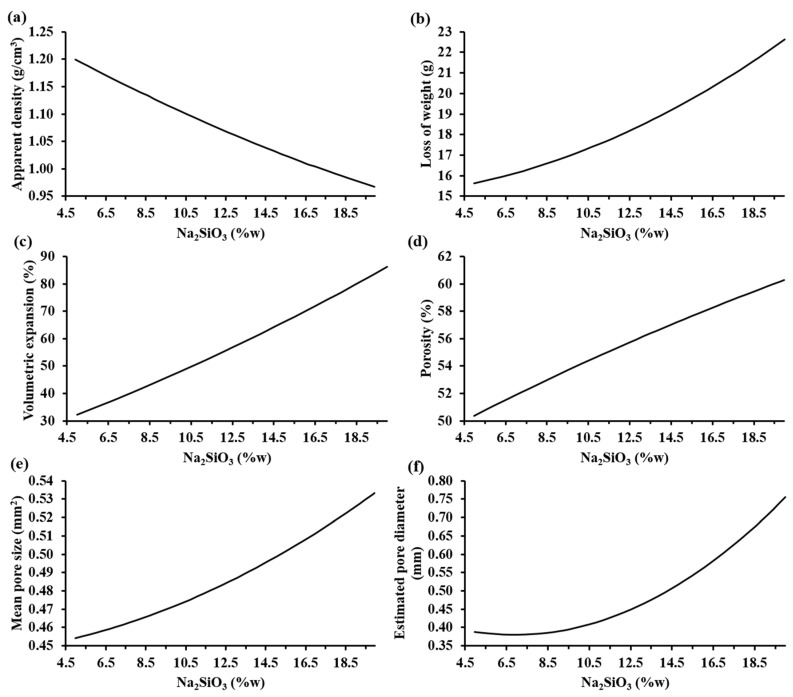
Trends of the characterization parameters as a function of the sodium silicate mass fraction according to MODDE® based on experimental results: (**a**) apparent density (SD: ±0.14 g/cm^3^), (**b**) loss of weight (SD: ±0.26 g), (**c**) volume expansion (SD: ±3.08%), (**d**) porosity (SD: ±2.13%), (**e**) mean pore size (SD: ±0.04 mm^2^), and (**f**) estimated pore diameter (SD: ±0.08 mm) of the foamed materials at 860 °C.

Figure 6 shows the trends of the characterization parameters as a function of the sodium silicate mass fraction according to MODDE® based on the experimental results. It can be observed that, when the sodium silicate content increases, both the loss of weight, volumetric expansion, porosity, mean pore size, and estimated pore diameter increased; meanwhile, the apparent density decreased. Specifically, the apparent density decreased to values lower than 0.95 g/cm^3^ and the porosity of the samples increased to values higher than 60% when the highest percentage of sodium silicate was employed. 

The influence of the increase in the sodium silicate was visible with a bigger pore generation that caused a higher volumetric expansion of the samples, reaching 90% of the initial volume of the compacted mixture. This allowed lightweight materials to be obtained. The estimated pore diameter of the samples increased to values higher than 0.75 mm. Hesky et al. (2015) found that this increment in the pore size is caused by the coalescence of the smaller pores to bigger ones [7]. On the other hand, the loss of weight of the samples was higher than 22% when the highest percentage of sodium silicate was used. This loss of weight is associated to the vaporization of the water from the aqueous solution of sodium silicate and the CO_2_(g), a product of the partial decomposition of Na_2_CO_3_(s) into Na_2_O(s) and CO_2_(g) [15]. 

**Figure 7:**
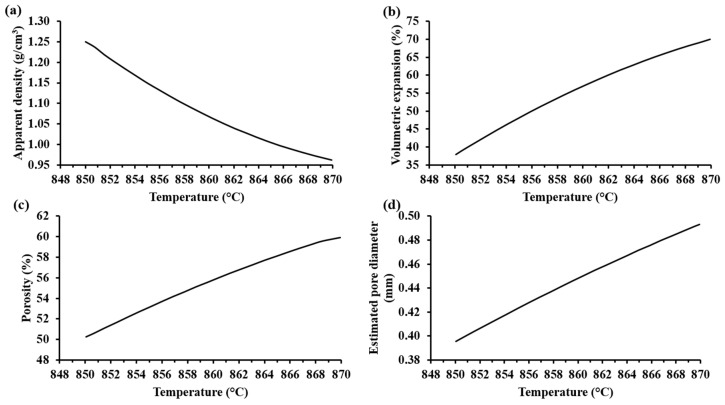
Trends of the characterization parameters as a function of the foaming temperature according to MODDE® based on experimental results: (**a**) apparent density (SD: ± 0.003 g/cm^3^), (**b**) volume expansion (SD: ± 23.04%), (**c**) porosity (SD: ± 0.04%), and (**d**) estimated pore diameter (SD: ± 0.27 mm) of the foamed materials with 20%w Na_2_SiO_3_(l).

Figure 7a shows that the apparent density of the samples decreased with an increase in the foaming temperature. Accordingly, the volumetric expansion, porosity, and pore diameter of the samples increased when the foaming temperature was increased (see Figure 7b–d). Visibly, the foamed samples at the highest temperature showed the biggest pores (see Table 5). Hesky et al. (2015), found that the cause of these bigger pores is the rapid liberation of the residue water from the hydrated silica molecules at higher temperatures (>800 °C), which generates a greater vapor pressure that provokes a more rapid pore expansion. This dehydration reaction begins at 700 °C [7]. Considering that the softening temperature of the SiO_2_-CaO-Na_2_CO_3_ mixture is around 850–900 °C, the pore formation in the samples is mainly due to the dehydration reaction of the hydrated form of silica. Thermal treatments at 850 °C for 30 min for compacted mixtures of SiO_2_-CaO-Na_2_CO_3_ did not show any appreciable porosity, suggesting that the CO_2_(g) produced by the decomposition of Na_2_CO_3_(s) is not responsible for the porosity of the foam samples. According to Kim and Lee (2001), the thermal decomposition of pure Na_2_CO_3_(s) (with a melting point at 850 °C) is too slow, and appreciable loss of mass product of the volatilization begins at 900 °C [15]. 

Through the optimizer tool of the software MODDE®, it was established that a mixture composition of 59.83%w SiO_2_(s), 16%w Na_2_CO_3_(s), 5.1%w CaO(s), and 19.99%w Na_2_SiO_3_(l) and a temperature of 859 °C are the optimum controlled factors to obtain a foamed material with the minimum apparent density and maximum porosity possible. The samples elaborated at these conditions presented an apparent density of 1.09 ± 0.03 g/cm^3^ and a porosity of 56.01% ± 1.12%. The mechanical strength for these samples was measured in a CBR loading press at a loading rate of ¼ inches per 30 s, resulting in 3.90 ± 0.16 MPa.

**Figure 8:**
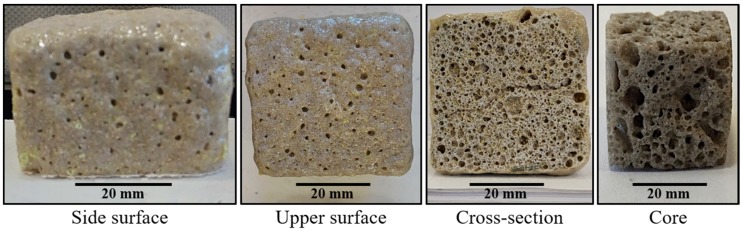
The cellular structure of the solid foam obtained using sodium silicate as a foaming agent. Material with a composition of 59.2%w SiO_2_(s), 16%w Na_2_CO_3_(s), 5.7%w CaO(s), and 19.1%w Na_2_SiO_3_(l).

The properties and, consequently, the possible applications of the cellular solids are a function of their cellular structure and porosity. As can be seen in Figure 8, some samples are mainly open-cell foams with a very wide distribution of the cell sizes, where the smaller cells make up the cell walls of the larger cells, giving a hierarchical cellular material with cells at several levels of scales [16]. Quartz is covalently bonded, giving the obtained ceramic foams higher melting points and moduli than other types of materials (e.g., polymer foams). These covalent bonds give strong elastic coupling between atoms too, which results in larger thermal conductivities [16]. However, the thermal conductivity of the porous materials is a function of the porosity, the diameter of the pores, and the kind of pores (open or closed) [17]. Glass and ceramics foams such as those obtained in this work become attractive as insulation materials due to their higher melting points [6,18]. Taking this into account, the process proposed in this work allows solid foams to be obtained with high melting points and with porosities and type of pores that produce materials that could find application as insulation materials. These kind of silica foams find application in the filtration of liquid metals and for gas and liquid phase catalysis at elevated temperatures due to their high melting points [16].

Commonly, an industrial glass foam insulation panel has a closed cellular structure with a mean pore diameter of 1.5 mm, average porosities of 80% and apparent densities <0.30 g/cm^3^ [1,19]. The production process of these glass-ceramic panels is through the mixing of glass powder with foaming agents usually from carbonaceous sources [16,17]. The focus of recent investigations on the development of glass-ceramic foams is the use of alternative raw material sources, such as waste-glass, flay ash, tailings, and other recycled industrial wastes [14]. The glass-ceramic foams obtained from these raw material sources present a wide range of characteristic properties. According to the literature, foams prepared using different proportions of waste-glass and other several components present mean cell dimensions of 0.25–2.00 mm, porosities of 60%–86%, and apparent densities of 0.13–0.8 g/cm^3^ [5,6,19,20,21]. Foams prepared using different proportions of fly ash and several other components present mean cell dimensions of 0.2–3 mm, porosities of 35%–78%, and apparent densities of 0.14–1.37 g/cm^3^ [22,23,24,25,26]. Foams prepared using different proportions of tailings and several other components present mean cell dimensions of 0.2–4.0 mm, porosities of 35%–84%, and apparent densities of 0.19–1.37 g/cm^3^ [8,9,10,24,25,26,27]. According to Table A1, the foams obtained in this work have characteristic properties within the ranges of the foams made with these alternative sources. However, the use of only quartz as a source of SiO_2_ brings the opportunity to use an alternative cheap source of silica that can be extracted from waste sources such as mine tailings. On the other hand, the use of sodium silicate as a foaming agent allows a powder foaming process without the emission of pollutants gases (e.g., CO_2_) harmful to the environment. The direct foaming process proposed in this work allows the production of silica foams at relatively lower temperatures and without the use of melting pots, which could be translated into cost reductions due to lower energy consumption, expenses in melting pots, and loss of foamed material.

## 4. Conclusions

The present study reveals that a direct foaming process of mixtures without melting pots of commercial quartz with CaO and Na_2_CO_3_ as fluxing agents and Na_2_SiO_3_·5H_2_O as a foaming agent allows the production of foams with different characteristics that could find applications as construction material and filter and catalysis material at high temperatures. The study of the minimum values of the temperature and time of the foaming process showed that foams with symmetric expansions and without fissures can be produced from a temperature of 850 °C, a minimum foaming time of 45 min, and a pressing pressure in a range of 4–6 t. The multilevel quadratic design realized with statistical software (MODDE®) showed that the foaming of the samples is principally affected by the temperature and the content of the sodium silicate. Their apparent density decreased with an increase in the concentration of Na_2_SiO_3_(l) and foaming temperature, and their porosity increased with an increase in the concentration of Na_2_SiO_3_(l) and foaming temperature. The samples obtained at the optimum controlled factors (59.83%w SiO2(s), 16%w Na_2_CO_3_(s), 5.1%w CaO(s), 19.99%w Na_2_SiO_3_(l) and a temperature of 859 °C for 45 min) showed an apparent density, porosity, and mechanical strength of 1.09 ± 0.03 g/cm^3^, 56.01% ± 1.12%, and 3.90 ± 0.16 MPa, respectively. Glass and ceramic foams such as those obtained in this work become attractive as insulation materials that could find applications where high temperatures occur due to their higher melting points. For instance, Hesky et al. [7] commented that thermal conductivity of porous materials depends on the heat transfer by conduction through the solid and gaseous phase and radiation. With decreasing the solid content of a material, porosity increases and the thermal conductivity decreases. In addition, with pore sizes higher than 5 mm, convection may occur and the thermal conductivity increases [28]. For the samples analyzed in our manuscript, the smallest pore dimension was 0.11 mm, and the largest pore dimension was 10.13 mm. Therefore, it is possible to assume that the samples with pores of a dimension lower than 5 mm could be used as insulation materials.

These kind of silica foams could find application in the filtration of liquid metals and for gas and liquid phase catalysis at elevated temperatures. Future research can be related to the use of SiO_2_ from alternative sources, such as tailings and other recycled wastes from mining in the preparation of ceramic foams.

## Figures and Tables

**Table 1:** Chemical composition of the pulverized quartz.

Element	(%)
Si	47.410
S	0.392
Cl	0.059
K	0.101
Ca	0.066
Ti	0.012
Cr	0.008
Fe	1.600
Cu	0.009

**Table 2:** Evaluated values to determine the minimums at which porosity and a symmetric expansion are produced.

Factors	Evaluated Values
Temperature (°C)	750, 800, 850
Time (min)	30, 45, 60
Pressing pressure (t)	2, 4, 6, 8, 10
Mixture composition:	
SiO_2(s)_ (%w)	59.2
Na_2_CO_3(s)_ (%w)	19.0
CaO_(s)_ (%w)	5.4
Na_2_SiO_3(l)_ (%w)	16.4

**Table 3:** Evaluated values to determine the minimums at which porosity and a symmetric expansion are produced.

Factors	Ranges and Fixed Values
Temperature (°C)	850–870
Time (min)	45
Pressing pressure (t)	4
Mixture composition:	
SiO_2(s)_ (%w)	59.20–70.3
Na_2_CO_3(s)_ (%w)	16.0–19.0
CaO_(s)_ (%w)	4.8–5.7
Na_2_SiO_3(l)_ (%w)	5.0–19.1

**Table 4:** Essay proposed by MODDE® in order to determine the minimums at which porosity and a symmetric expansion are produced.

Essay	T (°C)	SiO_2_ (%w)	Na_2_SiO_3_ (%w)	CaO (%w)	Na_2_CO_3_ (%w)
SS-01	850	59.20	19.10	5.70	16.0
SS-02		59.20	17.00	4.80	19.0
SS-03		70.30	5.90	4.80	19.0
SS-04		59.20	19.00	4.80	17.0
SS-05		59.20	16.40	5.40	19.0
SS-06		70.30	6.00	5.70	18.0
SS-07		70.30	8.60	5.10	16.0
SS-08		70.30	8.30	5.40	16.0
SS-09		62.90	16.30	4.80	16.0
SS-10		66.60	8.70	5.70	19.0
SS-11	870	59.20	20.00	4.80	16.0
SS-12		70.30	5.00	5.70	19.0
SS-13		70.30	8.90	4.80	16.0
SS-14		59.20	17.00	4.80	19.0
SS-15		59.20	16.10	5.70	19.0
SS-16		70.30	6.90	4.80	18.0
SS-17		70.30	7.00	5.70	17.0
SS-18		70.30	5.60	5.10	19.0
SS-19		62.90	13.30	4.80	19.0
SS-20		62.90	15.40	5.70	16.0
SS-21		66.60	11.70	5.70	16.0
SS-22		59.20	18.05	5.25	17.5
SS-23	860	64.75	12.50	5.25	17.5
SS-24		64.75	12.50	5.25	17.5
SS-25		64.75	12.50	5.25	17.5
SS-26		64.75	12.50	5.25	17.5

**Table 5:** Characterization chart for foams obtained using sodium silicate as a foaming agent.

Material	Quartz (Mainly)
Apparent density, $\rho_{app}$ (g/cm^3^)	0.66–1.73
Porosity, ϕ (%)	32.70–73.41
Volumetric expansion, $V_{e}$ (%)	−10.81–160.42
Mean pore diameter, $D_{p}$(mm)	0.18–1.58
Open or closed cells	Open and closed
Symmetry of cell structure	Axisymmetric

## Appendix A

**Table A1:** Results of characterization and pore structures of the foamed and sintered materials for 45 min using sodium silicate (Na_2_SiO_3_) as a foaming agent, varying the temperature and the mixture composition according the essay proposed using MODDE®.

Sample	T (°C)	SiO_2_ (%w)	Na_2_SiO_3_ (%w)	CaO (%w)	Na_2_CO_3_ (% w)	$\rho_{app}\;(g/{\mathrm{cm}}^{3})$	ϕ (%)	$V_{e}\;(\%)$	$D_{p}\;(\mathrm{mm})$	Pore Structures
SS-01	850	59.20	19.10	5.70	16.0	1.18	54.20	10.74	0.38	
SS-02		59.20	17.00	4.80	19.0	0.85	66.30	137.66	1.58	
SS-03		70.30	5.90	4.80	19.0	1.22	51.97	19.09	0.64	
SS-04		59.20	19.00	4.80	17.0	0.95	62.38	102.50	1.13	
SS-05		59.20	16.40	5.40	19.0	0.83	68.01	124.45	0.95	
SS-06		70.30	6.00	5.70	18.0	1.73	32.70	−10.81	0.21	
SS-07		70.30	8.60	5.10	16.0	1.54	40.00	−8.57	0.18	
SS-08		70.30	8.30	5.40	16.0	1.37	46.69	−5.71	0.27	
SS-09		62.90	16.30	4.80	16.0	0.95	61.44	56.62	0.48	
SS-10		66.60	8.70	5.70	19.0	1.43	43.04	12.88	0.29	
SS-11	870	59.20	20.00	4.80	16.0	0.80	68.38	160.42	1.39	
SS-12		70.30	5.00	5.70	19.0	1.10	56.98	30.07	0.42	
SS-13		70.30	8.90	4.80	16.0	1.19	53.01	22.77	0.37	
SS-14		59.20	17.00	4.80	19.0	0.85	66.24	105.08	1.20	
SS-15		59.20	16.10	5.70	19.0	0.77	70.08	79.30	0.69	
SS-16		70.30	6.90	4.80	18.0	0.92	63.87	62.85	1.13	
SS-17		70.30	7.00	5.70	17.0	1.07	57.27	25.89	0.39	
SS-18		70.30	5.60	5.10	19.0	0.94	62.86	70.27	0.55	
SS-19		62.90	13.30	4.80	19.0	0.66	73.41	134.38	0.56	
SS-20		62.90	15.40	5.70	16.0	0.81	67.99	100.89	0.43	
SS-21		66.60	11.70	5.70	16.0	1.00	60.64	40.63	0.29	
SS-22		59.20	18.05	5.25	17.5	0.79	69.22	126.85	1.12	
SS-23	860	64.75	12.50	5.25	17.5	1.20	52.66	42.05	0.40	
SS-24		64.75	12.50	5.25	17.5	0.95	52.17	35.51	0.53	
SS-25		64.75	12.50	5.25	17.5	1.19	52.94	42.05	0.34	
SS-26		64.75	12.50	5.25	17.5	1.26	50.91	39.87	0.42	
